# A Composite Analysis of Flowering Time Regulation in Lettuce

**DOI:** 10.3389/fpls.2021.632708

**Published:** 2021-03-08

**Authors:** Rongkui Han, Maria José Truco, Dean O. Lavelle, Richard W. Michelmore

**Affiliations:** ^1^The Genome and Biomedical Sciences Facility, University of California, Davis, Davis, CA, United States; ^2^Plant Biology Graduate Group, University of California, Davis, Davis, CA, United States; ^3^Department of Plant Sciences, University of California, Davis, Davis, CA, United States

**Keywords:** flowering time, genetic mapping, quantitative trait loci (QTL) mapping, genome-wide association study, circadian clock, lettuce, crop breeding

## Abstract

Plants undergo profound physiological changes when transitioning from vegetative to reproductive growth. These changes affect crop production, as in the case of leafy vegetables. Lettuce is one of the most valuable leafy vegetable crops in the world. Past genetic studies have identified multiple quantitative trait loci (QTLs) that affect the timing of the floral transition in lettuce. Extensive functional molecular studies in the model organism Arabidopsis provide the opportunity to transfer knowledge to lettuce to explore the mechanisms through which genetic variations translate into changes in flowering time. In this review, we integrated results from past genetic and molecular studies for flowering time in lettuce with orthology and functional inference from Arabidopsis. This summarizes the basis for all known genetic variation underlying the phenotypic diversity of flowering time in lettuce and how the genetics of flowering time in lettuce projects onto the established pathways controlling flowering time in plants. This comprehensive overview reveals patterns across experiments as well as areas in need of further study. Our review also represents a resource for developing cultivars with delayed flowering time.

## Introduction

Flowering plants dominate terrestrial landscapes and play a central role in the human food system. The timing of flowering is critical for the survival and adaptation of species to their environments because of the various ways a plant’s life cycle depends on the correct combination of external factors and internal signals, ranging from temperature, water availability, and pollinator activities, to the plants’ hormonal and carbon accumulation status. From the perspective of agricultural production, the timing of flowering often affects the quality and quantity of harvests. Stable flowering time has been a major objective for breeding endeavors because it provides a foundation for reliable crop production (Leijten et al., 2018). Shifting the flowering time of a crop has allowed breeders to introduce varieties adapted to new or changing growing conditions (Jung and Müller, 2009).

Due to its importance in both basic and translational biology, flowering time has been extensively studied in model plant species, particularly in *Arabidopsis thaliana.* In Arabidopsis, flowering time is controlled through an intricate network of genes that respond to diverse environmental stimuli and developmental signals. Externally, flowering time is primarily regulated by temperature and day length; internally, flowering time is regulated by gibberellic acid, carbohydrates, and age. Information from different pathways gets synthesized through key floral integrator genes, whose expression initiates the vegetative-to-floral identity shift of the apical meristem (Lee and Lee, 2010; Srikanth and Schmid, 2011). This network has been shown to be conserved across diverse angiosperms with various degrees of modification (Hecht et al., 2005; Abou-Elwafa et al., 2010; Luo et al., 2013; Liu et al., 2018a). This genetic and molecular research in Arabidopsis has provided a framework for understanding flowering time regulation in other plants.

Lettuce (*Lactuca sativa*) is one of the most popular leafy vegetables in the United States. With an annual domestic farm-gate value of more than $2.9 billion USD, lettuce was the highest-valued fresh-market vegetable in the country in 2019 (USDA-NASS., 2019). On a global scale, China is the world leader in lettuce production, producing four times as much as the United States and contributing to approximately half of the world’s lettuce production (FAO, 2020). Lettuce is harvested while it is in its vegetative growth state as it is consumed solely for its leaves in the US. The initiation of transitioning to reproductive growth is marked by the elongation of the stem, an event referred to as “bolting.” Bolting renders the crop bitter and unmarketable (Ryder, 1996). Therefore, delayed bolting and flowering is preferred in lettuce for maximizing harvestable yield while maintaining culinary quality; however, overly delayed flowering is unfavorable for seed production purposes. Lettuce is a self-pollinating plant whose flowering is accelerated under longer day lengths (Waycott, 1995) and higher ambient temperatures (Ryder, 1996). Wild lettuce (*L. serriola*), the wild progenitor of cultivated lettuce (Zhang et al., 2017), commonly exhibits a summer annual or winter annual growth habit. As a summer annual, wild lettuce seeds imbibe, germinate and flower rapidly under the long-day condition of spring and summer; as a winter annual, they germinate in the fall, overwinter as a vegetative rosette, and flower in the spring or summer in the following year. Vernalization, a period of cold treatment below a certain temperature threshold, is therefore required for some wild lettuce accessions to transition to reproductive growth (Prince et al., 1978). Understanding the genetic regulation of these biological processes as they related to flowering time in cultivated and wild lettuce is of great interest to biologists and breeders alike.

Over the past decade, numerous genetic and genomic resources have become available for lettuce. In 2013, an ultra-high density genetic map for lettuce was published (Truco et al., 2013); in 2017, a chromosome-scale lettuce reference genome assembly became available (Reyes-Chin-Wo et al., 2017). Multiple mapping populations have been developed and studied across the globe to investigate the genetic regulation of various agronomic traits of this internationally enjoyed vegetable (Silva et al., 1999; Hartman et al., 2013a,b; Truco et al., 2013). Sequencing of diverse accessions of wild and cultivated lettuce has enabled genome-wide association studies (GWAS) of commercially valuable traits (Kwon et al., 2013; Sthapit Kandel et al., 2020). Functional analyses of putative flowering time genes in lettuce have unveiled some of the molecular mechanisms underlying flowering time regulations. The latter approach is mostly enabled by the identification of lettuce orthologs to Arabidopsis flowering time genes through sequence similarity (Abbott, 2010; Fukuda et al., 2011, 2017; Han Y. et al., 2016a; Chen et al., 2018a,b).

The aim of this review is to consolidate current knowledge of the genetic and molecular control of flowering time in lettuce in the context of the current lettuce reference genome. In the Genetic Analysis section of this review, we collate information from all published and several unpublished genetic mapping experiments on bolting and flowering time in lettuce to compose a comprehensive list of the known quantitative trait loci (QTLs) segregating among cultivars and wild accessions. By anchoring flanking markers of these QTL on version 8 of the lettuce reference genome assembly, we provide a common framework for comparing and cross-referencing these QTLs as well as rationalizing their nomenclature. This broad view enables an appreciation of the genetic diversity underlying the phenotypic variation of flowering time in lettuce. Using sequence homology, we located putative orthologs of flowering time genes identified in Arabidopsis and compared their physical coordinates in reference to the bolting and flowering time QTLs in lettuce. In the Integration section of this review, we provide a detailed summary of the experimental evidence relating to the molecular mechanism of flowering time regulation in lettuce and organize these molecular experiments according to the pathways they pertain to. By comparing the assembled knowledge of flowering time regulation in lettuce to its well-studied counterpart in Arabidopsis, we were able to map conservation and divergence of the flowering time regulation pathways between the two species as well as identify underexplored areas and approaches that can potentially benefit the scientific and breeding communities.

## Materials and Methods

### Physical Location of Published QTLs

Physical coordinates of the markers flanking significant QTLs were reported in seven studies (Niroula, 2017; Mamo et al., 2019; Sandoya et al., 2020; Seki et al., 2020; Sthapit Kandel et al., 2020; Niroula et al., unpublished; You et al., unpublished). DNA variant detector array probe sequences of the lettuce expression sequence tag (EST)-based single nucleotide polymorphism (SNP) markers used in four studies (Hartman et al., 2013b; Jenni et al., 2013; Kwon et al., 2013; Niroula, 2017) were blasted against version 8 of the reference genome (Reyes-Chin-Wo et al., 2017; NCBI: GCA_002870075.2) to determine their physical locations. The coordinates of amplified fragment length polymorphism (AFLP) markers used in two studies could not be determined (Lavelle, 2009; Hartman et al., 2012, 2013b); therefore, the genetic locations of the peaks of the QTLs discovered in these studies were reported instead. QTL intervals of the GWAS were determined by extending the physical coordinates of flanking markers of significant peaks by 4 Mb on each side, according to the results of the linkage disequilibrium analysis in Kwon et al. (2013).

### Unpublished Studies

A population consisting of 97 F_8_ recombinant inbred lines (RILs) had been derived by single-seed descent from a cross between *L. sativa* cv. Salinas and *L. serriola* accession US96UC23 (Truco et al., 2013). This RIL population was planted in spring 2009 in an experimental field in Salinas, CA. Each experiment was arranged in a randomized complete block design with two blocks and was surrounded by guard rows. The plants were transplanted in 1 day. All plots were overhead-irrigated immediately after transplanting. Fertilization, pest control, and disease control were carried out according to standard protocols. The developmental stage of the plants was scored once a week. Four plants at the center of each plot were scored. The first scoring was on June 5th and the last scoring was on September 16th.

DNA from parental lines and the RILs were genotyped for 3,696 SNPs using the lettuce GeneChip genotyping protocol (Truco et al., 2013). Polymorphic markers were used to construct a genetic map using procedures described in Truco et al. (2013). A QTL analysis was conducted using composite interval mapping in QTL Cartographer 2.0 (Basten et al., 1994). Significance thresholds at *p* < 0.05 were calculated for each trait by permutation analysis with 1,000 permutations.

Two (Davis02 and Salinas04) out of the three trials using RILs of the Salinas × US96UC23 population reported by Lavelle (2009) were reanalyzed using the 3,696 lettuce GeneChip SNP markers, with composite interval mapping in QTL Cartographer 2.0 (Basten et al., 1994). Significance thresholds at *p* < 0.05 were calculated for each trait by permutation analysis with 1,000 permutations.

### QTL Name Rationalization

When necessary, QTLs were named or renamed according to the convention established for lettuce with the abbreviation of the associated phenotype and chromosomal linkage group preceded by the letter “q.” “FLT” was used for flowering time and “BLT” for bolting time. The numbering of QTLs on the same chromosome was assigned according to the time of publication. Priority was given to the earliest publication when the same QTL had been identified in more than one study or when the same designation had been used for different QTLs.

### Orthology

Orthofinder (Emms and Kelly, 2015) was used for genome-wide prediction of lettuce orthologs of flowering-time-related genes characterized in *A. thaliana*. Amino acid sequences of seven eudicot genomes, *A. thaliana*, *Solanum lycopersicum*, *Daucus carota*, *Helianthus annuus*, *Cichorium intybus*, *L. serriola*, and *L. sativa*, were used in the orthology analysis. The list of flowering time genes was obtained from the interactive database of flowering-time gene networks in *A. thaliana* (Bouché et al., 2015)^1^.

### Expression Analysis

RNA-seq reads from the time course experiment described in Higashi et al. (2016) were quality and adapter trimmed using BBDuk^2^ and mapped to version 8 of the lettuce reference genome (Reyes-Chin-Wo et al., 2017) using STAR (Dobin et al., 2013). Oscillation of transcription level (in reads per million mapped reads) and period of oscillation were detected using R package DiscoRhythm (Carlucci et al., 2019). Transcripts with *p* < 0.05 over a 24-h oscillation period are reported.

## Genetic Analysis

A systematic literature review and consultation with the research community identified nine published studies (Hartman et al., 2012, 2013a,b; Jenni et al., 2013; Kwon et al., 2013; Mamo et al., 2019; Sandoya et al., 2020; Seki et al., 2020; Sthapit Kandel et al., 2020), two dissertations (Lavelle, 2009; Niroula, 2017), and four unpublished datasets (Niroula et al., unpublished; You et al., unpublished; Han et al., unpublished; M.-J. Truco, unpublished) pertinent to this study. These data represent a comprehensive collection of genetic mapping studies of flowering time and bolting time in lettuce. A total of 56 field and greenhouse experiments have been conducted between 2002 and 2019, testing 11 mapping populations (for QTL mapping) and 2 diversity panels (for GWAS). Data including the parental lines and generation of the mapping populations, the number of lines, the type and number of markers, mapping software, and parameters used in the analysis, as well as the time and location of each experiment are summarized in Supplementary Table 1. Most experiments tracked either the flowering time (23) or bolting time (13) phenotype, while 20 studies tracked both. Over three quarters (41) of the experiments were conducted in the field. The geographic locations of experiments included Chile, Japan, the Netherlands, southeastern Canada, and West Coast of the United States. These growing areas differ substantially in temperature and humidity. Notably, however, nearly all experiments (50) were conducted under long-day (LD) conditions with photoperiods longer than 12 h per day. Only three mapping populations have been tested in a total of six experiments under both LD and short-day (SD) conditions.

**FIGURE 1:**
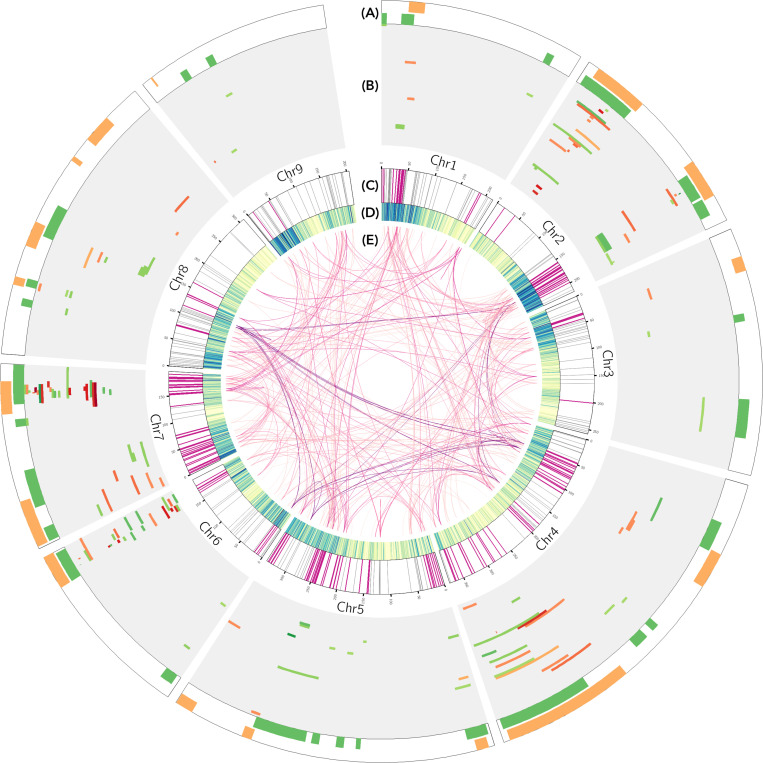
**(A)** Physical location of 35 bolting time (green) and 32 flowering time (yellow) consensus quantitative trait loci (QTLs) in lettuce. **(B)** Physical location of 167 QTLs reported in a total of 56 field and greenhouse experiments. Each track represents one experiment. Earlier reported experiments are in the outer tracks. Bolting time QTLs are indicated by green color blocks and flowering time QTLs by orange blocks. Saturation of the color blocks indicates percent phenotypic variance explained by the QTLs. The physical locations of 12 QTLs from three experiments (Summer 2003 from Lavelle, 2009 and both experiments from Hartman et al., 2013b) could not be located on the genome because amplified fragment length polymorphism (AFLP) markers were used. **(C)** Location of lettuce orthologs of genes with flowering time function in Arabidopsis. Flowering time orthologs within known QTLs are highlighted in fuchsia. **(D)** Gene density of the lettuce genome. **(E)** Flowering time orthologs within the same orthogroup are connected. Darker shade connections indicate larger orthogroups.

A total of 167 QTLs have been reported for bolting and flowering time in lettuce. Merging QTLs with extensively overlapping intervals reduces this number to 67. Bolting and flowering time QTLs are located on all nine lettuce chromosomes (Figure 1). Chromosomes 2 and 7 have the highest counts of QTLs. Specifications of each QTL including physical and genetic locations as well as effect sizes in each experiment are provided in Supplementary Table 2. Seven major QTLs each explained more than 30% of phenotypic variance in their respective experiments. The effect of any one QTL can be highly variable across trials. For instance, *qFLT7.1* explained 11.2, 30.23, 39.6, and 51.7% of phenotypic variance in separate experiments that used the same mapping population, PI251246 × Salinas. The vast majority of the reported QTLs are environmentally sensitive; their effects on the phenotype only manifested in a subset of the experiments that used a specific mapping population. This is in line with the current understanding of the mechanism of flowering time regulation as one that integrates both internal and external signals. It is possible that the environment-sensitive QTLs represent genetic variations in the upstream signaling of the flowering time pathway, where environmental cues are perceived and transduced into molecular signals, while QTLs that are consistently detected across all environments represent variation in downstream components that affect flowering time regardless of external cues. Some QTLs identified under SD conditions overlapped with QTLs identified under LD conditions in the same mapping populations (*qBLT6.1*, *6.5* and *qFLT2.1*, *4.3*, and *6.1*), while other SD QTLs were novel (*qFLT1.2*, *4.5*, and *9.5*); all novel SD QTLs were detected in the Armenian × PI251246 population (Han et al., unpublished). The detection of novel SD QTL suggests this population segregates for photoperiod sensitivity; and photoperiod sensitivity is regulated separately from daylength-independent flowering time. Fifteen of the 67 QTLs were discovered in multiple populations that do not share parents; nevertheless, the parental lines could share regions of identity by descent. All other QTLs were identified in only one mapping population. These results suggest that modification of flowering time phenotype is achieved through a variety of strategies in different lettuce cultivars and wild lettuce accessions as in other species. The lack of overlapping results in past studies also suggests that there are likely to be additional polymorphic loci regulating flowering time that remain to be discovered.

About half (35) of the reported QTLs are for the bolting time phenotype, despite the over-representation of flowering time being the primary phenotype of interest. Nine QTLs have a pleiotropic effect on both bolting time and flowering time as shown by their colocation on the lettuce genome (Figure 1). The low frequency of collocation between bolting and flowering time QTLs can be partly explained because most studies only tracked one of the two phenotypes. Nevertheless, this might also reflect genetic differences where the pathways controlling bolting and flowering are distinct, thus providing opportunities for understanding the transition between stem elongation and floral initiation.

## Integration of Functional Information From Model Systems

Adjustment of flowering time based on environmental and internal cues is realized via the intricate interplay of seven major genetic pathways in Arabidopsis. These include the vernalization pathway, the autonomous pathway, the ambient temperature pathway, the photoperiod pathway, the hormone pathway, the aging pathway, and the sugar pathway (Srikanth and Schmid, 2011). Extensive research in *A. thaliana* has revealed the identity and function of pivotal genes in these pathways. We review these pathways and their key genic components in *A. thaliana*, followed by a summary of functional analyses that have been performed on their orthologs in lettuce. All pathways contain genes that collocate with known QTLs. The number of QTLs each pathway intersects with is relatively proportional to the number of genes present in the pathway. By contrasting our knowledge of flowering time regulation in lettuce to its counterpart in Arabidopsis, we aim to identify key differences between the two systems and provide data-informed suggestions for future breeding and scientific endeavors.

An analysis of orthology placed 306 Arabidopsis flowering time genes into 222 orthogroups; 237 of these genes have orthologs in lettuce (Supplementary Table 3). Four hundred and five lettuce genes were identified as flowering time orthologs representing 217 out of the 222 orthogroups (Table 1).

**TABLE 1:** Summary statistics of lettuce and Arabidopsis genes within each flowering time regulatory pathway.

**Pathways**	**Genes in lettuce**	**Genes in Arabidopsis**
Vernalization and autonomous	30	27
Ambient temperature	5	7
Photoperiodism and circadian clock	171	110
Gibberellic acid	37	28
Aging	18	22
Sugar	24	9
Floral integrator	17	8
Flower development and meristem identity	25	9
General	168	117
Two pathways	48	73
Three pathways	17	18
Four pathways and above	2	3

The majority of the 405 orthologs have at least two paralogs within the lettuce genome. Only 110 Arabidopsis flowering time genes have single lettuce orthologs. The largest flowering time orthogroups, which contains Arabidopsis *FT-INTERACTING PROTEIN 1* (*FTIP1*), has eight orthologs in the lettuce genome. The lettuce genome has undergone duplication and triplication events (Reyes-Chin-Wo et al., 2017); however, locations of paralogs within flowering time orthogroups is not obviously consistent with the triplication signature basal to the Compositae family (Figure 1; Reyes-Chin-Wo et al., 2017). The lack of correspondence to the triplication event may reflect gene expansion in earlier duplication events. Nearly half of the orthologs (190/405) lie within the known QTLs, while the rest do not. This supports the idea that there is undiscovered natural genetic variation for bolting and flowering time phenotypes. Conversely, four out of the 64 QTLs do not have putative orthologs within their intervals. Under 12 h light/12 h dark conditions and in 3-week-old vegetative plants, 43 orthologs show oscillating expression following 24-h periods (Supplementary Table 3). Twenty-five out of the forty-three oscillating transcripts are putative members of the photoperiod pathway and/or the circadian clock. Their oscillation provides support for the function of these genes in pathways that involve light sensing and time tracking.

There have been two genome-wide expression experiments that capture the transition between vegetative and reproductive growth in lettuce (Chen et al., 2018a,b; Liu et al., 2018b). One experiment compared heat-treated bolting lettuce with non-heat-treated rosette samples and identified 2,149 differentially expressed genes (DEGs; Liu et al., 2018b). The other experiment was performed in laser capture micro-dissected apical meristematic tissues (Chen et al., 2018a,b). This study reported 21 DEGs at the initiation of bolting (formation of dome-shaped apical meristem) and 365 DEGs after the apical meristem committed to reproductive growth (elongation of apical meristem; Chen et al., 2018a). The same group later used the same dataset to analyze the expression profile of 15 putative flowering-time orthologs in lettuce (Chen et al., 2018b); 4 showed upregulation at the initiation of bolting while 7 showed upregulation after the apical meristem committed to reproductive growth. Several of these key regulatory flowering-time orthologs are discussed in detail below according to the regulatory pathway they belong to.

### Vernalization and Autonomous Pathway

The vernalization and autonomous pathways are often discussed together because of the central role of *FLC* in both of them. Vernalization refers to the requirement of exposure of imbibed seeds or vegetative seedlings to a prolonged period of cold temperature to induce flowering. In Arabidopsis, vernalization is realized through *FRIGIDA* (*FRI*) and *FLOWERNG LOCUS C* (*FLC*). Naturally occurring mutations in *FRI* have been associated with the loss of the vernalization requirement in summer annual Arabidopsis accessions (Johanson et al., 2000). *FRI* is an up-regulator of *FLC* (Geraldo et al., 2009), which encodes a MADS box protein that directly represses flowering time genes *FD, FLOWERING LOCUS T* (*FT*), and *SUPPRESSOR OF OVEREXPERSSION OF CONSTANS 1* (*SOC1*; Searle et al., 2006). The expression of *FLC* is suppressed by gene *VERNALIZATION INSENSITIVE 3* (*VIN3*) and two antisense long RNAs of *FLC* itself, *COOLAIR* and *COLDAIR* (Sung and Amasino, 2004; Heo and Sung, 2011). The maintenance of the silencing of *FLC* is carried out through *VERNALIZATION 1* and *2* (*VRN1* and *VRN2*; Gendall et al., 2001; Levy et al., 2002). In contrast to vernalization pathway mutants, those in the autonomous pathway are characterized by delayed flowering regardless of environmental conditions. Genes on the autonomous pathways, including *FLOWERING CONTROL LOCUS A* (*FCA*), *FLOWERING LOCUS D* and *K* (*FLD* and *FLK*), *FPA*, *FY, LUMINIDEPENDENS* (*LD*), *FVE*, and *RELATIVE OF EARLY FLOWERING 6* (*REF6*), modulate *FLC* expression by either changing the chromatin configuration or participating in mRNA modification (Simpson, 2004).

Many accessions of wild lettuce (*L. serriola*) require vernalization to transition to reproductive growth. Early studies identified 10°C to be the upper limit for vernalizing wild lettuce (Warne, 1947). The response is quantitative, with longer cold-treatment periods associated with more accelerated flowering after the treatment. Imbibed seeds and older vegetative seedlings were both more responsive to vernalization treatment than cotyledon-stage seedlings (Warne, 1947; Prince et al., 1978). Vernalization can accelerate flowering in some cultivars of lettuce (*L. sativa*), such as the crisphead cultivar “Great Lake” (Rappaport et al., 1956), but not others such as the leafy cultivars studied by Zhang et al. (2016).

No lettuce ortholog of *AtFLC* has been identified, despite numerous attempts using multiple bioinformatic and molecular approaches (Reeves et al., 2007; Lavelle, 2009; Abbott, 2010), suggesting a potentially different molecular mechanism underlying vernalization in lettuce. *FLC* belongs to a distinct clade within the MLKC-type MADS box gene family (Hileman et al., 2006). The presence and function of genes in the *FLC* clade have been challenging to predict across eudicot lineages regardless of the evolutionary relationship between the species of interest (Becker and Theißen, 2003; Hecht et al., 2005; Hileman et al., 2006). Searches for *FLC* homologs have been made in legumes (Fabaceae; rosid), sunflower (*Helianthus annuus*; asterid), and tomato (*Solanum lycopersicum*; asterid), but with negative results (Hecht et al., 2005; Hileman et al., 2006; Blackman et al., 2011b). Nevertheless, an *FLC* homolog with conserved functions was identified in sugar beets (*Beta vulgaris*; Reeves et al., 2007), a member of the Caryophyllales more closely related to lettuce (asterid) than Arabidopsis (rosid). An *FLC*-like gene, *CiFL1*, has also been cloned in chicory (*Cichorium indybus*), a biennial Compositae species closely related to lettuce; expression analysis suggested it functions as a floral suppressor during the vernalization process in chicory (Périlleux et al., 2013). Orthologs of many other genes that function upstream of *FLC* in Arabidopsis have been identified and cloned in lettuce including *FCA*, *FLD*, *FLK*, *FPA*, *FY*, *LD*, and *FVE*. The identity between the amino acid sequence of the lettuce genes and their respective Arabidopsis orthologs ranges from 43 to 79% (Abbott, 2010). Among these genes, *LsFVE* exhibits peak expression before the vegetative–reproductive transition; maximum expression of *LsFLD* and *LsLD* overlapped temporally with the vegetative–reproductive transition period (Fukuda et al., 2017), suggesting the important role of the autonomous pathway in flowering time regulation of cultivated lettuce. Our orthology analysis identified a potential lettuce ortholog for *FRI* on Chromosome 5, Lsat_1_v5_gn_5_9321 (Supplementary Table 3); no molecular analyses have been conducted to study its function. The elusive evolutionary history of the *FLC* gene clade and the unresolved regulatory mechanism of vernalization in lettuce creates an interesting opportunity for future genetic and molecular studies.

### Ambient Temperature Pathway

High ambient temperature accelerates flowering in Arabidopsis (Balasubramanian et al., 2006). Although the regulatory mechanisms of ambient temperature influences on flowering time is not as fully elucidated as some of the other pathways, several genes that play key roles in this thermo-sensory response have been identified. The loss of *SHORT VEGETATIVE PHASE* (*SVP*) function resulted in insensitivity to ambient temperature changes and plants deficient in *ACTIN RELATED PROTEIN 6* (*ARP6*) display constitutive warm temperature responses. *SVP* is a MADS-box protein that binds to *FT* and *SOC1* promoters and acts as a repressor. It also mediates the temperature-dependent functions of *FCA* and *FVE* in the autonomous pathway (Lee et al., 2007). Chromatin immunoprecipitation (ChIP) analysis suggests that *SVP* may function in an *FLC*-dependent manner (Li et al., 2008). *ARP6* functions by introducing into nucleosomes, rather than H2A, a special histone H2A.Z, whose DNA wrapping capacities exhibits temperature dependency (Kumar and Wigge, 2010). There is extensive crosstalk between the ambient temperature and the vernalization/autonomous pathways. *FLC* is a potent suppressor of thermo-induced flowering, while *FLOWERING LOCUS M* (*FLM*), a MADS-box protein with extensive sequence similarity to *FLC*, co-locates with a QTL modulating thermosensitivity (Balasubramanian et al., 2006).

Heat-accelerated bolting and flowering is a common phenomenon that impacts agricultural production of diverse lettuce cultivars. Cultivars exhibit broad variation in their bolting behavior in response to high ambient temperature, making this trait an important subject of genotype × environment studies (Hwang et al., 2007; Jenni and Yan, 2009; Jenni et al., 2013; Han Y.Y. et al., 2016b; Lafta et al., 2017; Holmes et al., 2019). Intersecting QTLs with physical coordinates of lettuce flowering time orthologs suggests Lsat_1_v5_gn_2_93321, a MADS-box gene, might function in heat-induced bolting (Jenni et al., 2013). No definitive lettuce ortholog of *SVP* has been identified; this is at least partly due to the sequence-level similarity between *SVP* and *AGAMOUS-LIKE 24* (*AGL24*), a floral meristem identity gene that primarily functions downstream of the floral initiation pathway (Gregis et al., 2008). Four putative orthologs of *SVP* and *AGL24* are present in the lettuce genome; one of them, Lsat_1_v5_gn_3_62800, has been considered either an ortholog of *SVP* or *AGL24* (Supplementary Table 3; Huo et al., 2016; Chen et al., 2018b). Another ortholog of *SVP* and *AGL24*, Lsat_1_v5_gn_6_105061 (Supplementary Table 3), is located within *qFLT6.2* but has yet to be studied at the molecular level. The lack of clear lettuce orthologs of *FLC* and *SVP* has made it difficult to form testable hypotheses regarding the regulatory mechanism through which ambient temperatures accelerate bolting in lettuce. In addition, two studies have reported binding motifs of heat shock transcription factors in the promoter regions of *LsSOC1* and *LsMADS55*, putative lettuce orthologs to Arabidopsis *SOC1* and *APETALA1* (*AP1*), both known to function downstream of *FLC* in the floral induction pathway (Chen et al., 2018b; Ning et al., 2019). These reports postulate the possibility that the thermal control of flowering time in lettuce might have a different and more concise regulatory architecture than its counterpart in Arabidopsis. Exploratory studies using differential expression and protein accumulation analyses have suggested genetic elements actively involved in this process. Expression levels of lettuce *HEAT SHOCK PROTEIN 70* (*HSP70*), an output of the ambient temperature sensing pathway in Arabidopsis (Kumar and Wigge, 2010), differ between heat sensitive and heat tolerant lettuce lines, suggesting its conserved function in temperature sensing in lettuce (Li et al., 2017). A differential expression analysis comparing the gene expression profiles of heat-treated (bolted) and control (non-bolted) lettuce plants on the seventh day after the initiation of heat treatment revealed significant changes in *C2H2* zinc finger, *Ap2-EREBP*, and *WRKY* transcription factor families, indicating their potential functions in heat-induced bolting (Liu et al., 2018b). The same study also identified increased gibberellic acid (GA) and indole-3-acetic acid (IAA) prior to heat induced bolting. Two comparative proteomic studies have investigated differential protein accumulation in heat-treated lettuce plants. One study compared the heat response of an early bolting lettuce genotype with that of a late bolting genotype (Han Y. et al., 2016a) and reported elevated accumulation of metabolism-related proteins in the early bolting genotype and protein synthesis-related proteins up regulated in the late bolting genotype. The other compared heat-treated, bolted lettuce plants with non-treated, unbolted plants of the same genotype (Hao et al., 2018); this reported enrichment of proteins is associated with photosynthesis, tryptophan metabolism, and IAA biosynthesis. Additional studies are needed to disentangle the molecular signal of the bolting process from that of the thermo-sensory pathway that precedes bolting.

### Photoperiod Pathway

Arabidopsis is a facultative long-day plant, for which floral transition is promoted by LD conditions and is delayed, but not completely inhibited, by SD conditions (Mouradov et al., 2002; Fornara et al., 2010; Srikanth and Schmid, 2011). The photoperiodic control of flowering in Arabidopsis is realized through the integration of transcriptional and post-transcriptional regulations of a zinc finger transcription factor, *CONSTANS* (*CO*). The baseline expression level of *CO* is circadianly entrained, resulting in a 24-h-phase oscillation of its transcription (Suárez-López et al., 2001). This phasing is further modified by the *GIGANTEA–FLAVIN-BINDING, KELCH REPEAT, F-BOX 1–CYCLING DOF FACTOR 1* (*GI-FKF1-CDF1*) protein triad, resulting in a second peak of *CO* expression toward the end of the day under LD conditions (Imaizumi et al., 2005; Sawa et al., 2007; Fornara et al., 2009). At the post-transcriptional level, the protein stability of *CO* is regulated by the E3 ubiquitin ligase *CONSTITUTIVELY PHOTOMORPHOGENIC 1* (*COP1*) and members of the *SUPPRESSOR OF PHYA-105* (*SPA*) protein family in a daylength-dependent fashion (Hoecker and Quail, 2001; Laubinger et al., 2006; Liu et al., 2008b). Other systemic regulators of *CO* include *PHYTOCHROME B* (*PHYB*), *PHYC*, and *DAY NEUTRAL FLOWERING* (*DNF*) (Monte et al., 2003; Morris et al., 2010; Golembeski and Imaizumi, 2015). The expression of *CO* regulates flowering by promoting the production of a floral induction signal in leaves (Ayre and Turgeon, 2004). The signaling protein and mRNA encoded by *FT* are both transported to the shoot apical meristem via phloem companion cells to initiate flowering (Kardailsky et al., 1999; Weigel et al., 2000; Corbesier et al., 2007; Li et al., 2011). During vegetative growth, the expression of *FT* is repressed by histone trimethylation mediated by polycomb repressive complexes (Jiang et al., 2008).

Like Arabidopsis, lettuce is also a facultative long-day plant (Sukprakarn, 1985; Waycott, 1995). Lettuce exhibits an array of responses to changes in photoperiod, varying from the less responsive summer cultivars, such as the North American crispheads “Empire” and “Salinas,” to the highly sensitive winter cultivars, such as the European butterheads “May King” and “Saffier,” whose flowering time is significantly delayed as daylength shortens. However, the genetic determinants underlying the photoperiodic response have not been well-characterized. Three populations have been grown under both LD and SD conditions; the SD experiments detected nine QTLs; three of these SD QTLs were unique to the SD condition (*qFLT1.2*, *4.5*, and *9.*5; Supplementary Table 2). The causal genes underlying these QTLs are candidates for key elements of the photoperiod sensing pathway in lettuce.

Six *CO*-like genes have been identified in the lettuce genome (Supplementary Table 3). Two of these genes, Lsat_1_v5_gn_2_86121 and Lsat_1_v5_gn_5_122401, show oscillating expression in 24-hour periods. Lsat_1_v5_gn_5_122401 is within *qFLT5.3* (Seki et al., 2020). No *CO*-like gene has been analyzed functionally in lettuce. Consequently, the mechanism of the photoperiod control of flowering time in lettuce remains unresolved. Multiple lettuce genes may jointly fulfill the functions of *AtCO*; complementary functions have been reported for *CO*-like genes in sugar beet (*B. vulgaris*; Dally et al., 2018). A functional *CO* ortholog, *HaCOL2*, has been identified in sunflower (*H. annuus*), a close relative to lettuce; overexpression of *HaCOL2* complemented the Arabidopsis *co* mutation (Blackman et al., 2011a).

### Gibberellic Acid Pathway

In Arabidopsis, gibberellic acid (GA) acts as a flower promoting agent in parallel to *CO* activities; deficiency in GA biosynthesis has an additive effect on the late flowering phenotype of *co* mutants in LD (Putterill et al., 1995). GA functions by targeting DELLA proteins, which function as repressors of plant growth and development (Harberd et al., 2009; Sun, 2010), for ubiquitination, thereby promoting the expression of the floral integrators *SOC1* (Liu et al., 2008a) and *LEAFY* (LFY; Gocal et al., 2001; Achard et al., 2004).

Foliar application of exogenous GA induces bolting in lettuce (Kato, 1964b; Han Y. et al., 2016a) and endogenous GA may play a role in the ambient temperature signaling pathway in lettuce (Kato, 1964a). Under natural growing conditions, GA is barely detectable in lettuce apical meristems during vegetative growth; however, its concentration increases rapidly after floral induction (Kato, 1964a). After heat treatment, GA begins to accumulate shortly after treatment, resulting in a sharp increase similar to the GA accumulation observed during the natural bolting process (Kato, 1964a). This result has been confirmed using mass spectrometry to measure GA levels; in addition, the expression of *LsGA3ox1*, a GA 3-oxidase gene that metabolizes GA_20_ to the bioactive form GA_1_, is significantly upregulated by high temperature (Fukuda et al., 2009). Coupling of GA to the expression of major flower induction genes, such as *LsSOC1* and *LsLFY*, has yet to be demonstrated.

### Aging Pathway

Before transitioning to reproductive growth, the vegetative growth of a plant can be temporally divided into a juvenile phase and an adult phase (Huijser and Schmid, 2011). Vegetative phase change is controlled primarily by the interplay of two microRNAs (miRNAs), *miR156* and *miR172* (Wang et al., 2011). In Arabidopsis, *miR156* is highly abundant in juvenile seedlings; it represses flower induction by negatively regulating *SQUAMOSA PROMOTER BINDING PROTEIN LIKE* (*SPL*) genes. Both in concert and parallel with *FT*, *SPL* genes promote the expression of floral integrator genes *SOC1* and *LFY*, as well as the floral identity genes *FRUITFUL* (*FUL*) and *AP1* (Wang et al., 2011). *MiR172* is a direct target of *SPL9* in Arabidopsis (Wu et al., 2009); in contrast to miR156, its expression level gradually increases as the plant ages and the activity of the *SPL* genes rise. *MiR172* targets flower suppressors, such as *AP2*, *SCHLAFMÜTZE* (*SMZ*), *SCHNARCHZAPFEN* (*SNZ*), *TARGET OF EAT1* (*TOE1*), *TOE2*, and *TOE3*, for silencing (Schmid et al., 2003; Mathieu et al., 2009). This pathway is conserved across divergent monocot and dicot lineages (Lauter et al., 2005).

*LsMIR156* and *LsMIR172*, genes that encode *miR156* and *miR172* in lettuce, have been cloned and ectopically expressed in Arabidopsis. Consistent with the function of their respective orthologs in Arabidopsis, *LsMIR156* delayed flowering and *LsMIR172* accelerated flowering when overexpressed. In addition, the mRNA levels of *LsSPL3* and *4* were inversely correlated with that of *miR156* and positively with *miR172*, consistent with conservation of the aging pathway between Arabidopsis and lettuce. Interestingly, unlike the case in Arabidopsis, the function of *MIR156* in lettuce depends on the gene *DELAY OF GERMINATION1* (*DOG1*). In lettuce, *DOG1* has a pleiotropic effect on both seed germination and flowering time, while the Arabidopsis *dog1* loss-of-function mutant exhibits deficient seed dormancy but no flowering time phenotype (Huo et al., 2016).

### Sugar Pathway

The integration of carbohydrate status into the flowering time pathway is still poorly understood and the effect of sugars on flowering time varies across plant species (Srikanth and Schmid, 2011). In Arabidopsis, extremely high or low concentrations of sucrose delay flowering, while a 1% concentration of sucrose promotes earlier flowering (Ohto et al., 2001). Homeostasis of another sugar, trehalose, is also essential for the normal transition toward reproductive growth (Dijken et al., 2004; Paul et al., 2008).

The effects of sugar on flowering time have not been studied in lettuce. Orthologs of *ADG1* (Eimert et al., 1995), *HXK1* (Moore et al., 2003), *SUS4* (Seo et al., 2011), and *TPS1* (Dijken et al., 2004) on the sugar pathway in Arabidopsis are found within lettuce bolting and/or flowering time QTLs; however, orthologs of other flowering time genes were also present within these QTLs, making it unclear whether variations in the sugar pathway were involved in the phenotypic variations (Supplementary Table 3).

### Floral Integrators

FT, SOC1, and LFY are often referred to as floral integrators because of their pivotal role in connecting various floral induction pathways to the floral development pathway (Lee and Lee, 2010; Srikanth and Schmid, 2011). Experimental evidence puts the three genes along a signaling pathway from upstream to downstream in the order of *FT*, *SOC1*, and *LFY* (Yoo et al., 2005; Lee et al., 2008), each promoting the expression of its target. *FT* is directly targeted by *FLC*, *SVP*, and *CO*, thereby integrating signals from the vernalization/autonomous pathway, temperature pathway, and photoperiod pathway. *SOC1* is regulated by *FLC*, *SVP*, and GA signaling. *LFY* is regulated by GA and miRNAs (Yamaguchi et al., 2009; Wang et al., 2011). Through this network of interactions, the floral integrator genes are expressed under conducive environmental conditions, resulting in the upregulation of flower homeotic genes such as *AP1*, *APETALA3* (*AP3*), and *AGAMOUS* (*AG*), triggering the transition from vegetative to reproductive growth (Liu et al., 2009).

Lettuce orthologs of *FT* (Lsat_1_v5_gn_2_17881), *SOC1* (Lsat_1_v5_gn_7_6780), and *LFY* (Lsat_1_v5_gn_4_84380) have been identified and cloned (Abbott, 2010; Fukuda et al., 2011, 2017; Chen et al., 2018a,b). *LsFT* is located within *qFLT2.4* and *qFLT2.6*, while *LsSOC1* is located within *qFLT7.3* (Supplementary Table 3). As expected, none of these genes are expressed in vegetative lettuce plants.

*LsFT* plays an important role in the flowering time pathway in lettuce. Lettuce has only one identifiable *FT* ortholog, located on Chromosome 2, unlike in sunflower, where three *FT* orthologs have been identified (Oda et al., 2004; Blackman et al., 2011a; Fukuda et al., 2011; Reyes-Chin-Wo et al., 2017). The amino acid sequence of *LsFT* is 76% identical to *AtFT* (Abbott, 2010; Fukuda et al., 2011). Ectopic expression of *LsFT* using the 35S promoter induces early flowering in wild type Arabidopsis and partially rescues the late flowering phenotype of *ft-2* mutant Arabidopsis (Fukuda et al., 2011; Chen et al., 2018a). The expression level of *LsFT* increases during the transition from vegetative to reproductive growth; it is also up-regulated during heat-induced flowering (Fukuda et al., 2017). RNAi knockdown of *LsFT* delays bolting and diminishes bolting in response to heat treatment. Knockdown lines also show reduced expression of *LsAP1*, *LsAP3*, and *LsLFY*, suggesting that similar to their orthologs in Arabidopsis, these genes function downstream of *FT.* Early bolting lettuce cultivars exhibit higher endogenous *LsFT* expression levels than late bolting lines (Chen et al., 2018a).

*LsSOC1*, which encodes a MADS box transcription factor, is located on Chromosome 7. There are two other paralogs of *LsSOC1* on Chromosomes 3 and 4; however, genetic mapping (Hartman et al., 2013b) and molecular analysis (Chen et al., 2018b) both indicate that Lsat_1_v5_gn_7_6780 is the functional ortholog. *LsSOC1* expression increased in both heat-treated and non-heat-treated bolting lettuce plants. RNAi knockdown of *LsSOC1* resulted in delayed flowering and insensitivity to heat treatment in terms of bolting. Binding motifs of heat shock transcription factors have been identified in the promoter of *LsSOC1*, suggesting that *LsSOC1* might function in the ambient temperature pathway in lettuce (Chen et al., 2018b). However, the lack of a heat response in RNAi-*LsFT* lines suggests that *LsFT* might function as the end point of the ambient temperature pathway upstream of *LsSOC1*. Further molecular analyses are needed to clarify the regulatory relationship between *LsFT* and *LsSOC1*.

Unlike *LsFT* and *LsSOC1*, *LsLFY* is not located within any known QTLs (Supplementary Table 2). *LsLFY* encodes a transcription factor and is located on Chromosome 4. Under flowering-promoting conditions, its expression is upregulated in a synchronized fashion with the progression of floral development in field-grown lettuce, consistent with a role in flower bud formation (Fukuda et al., 2017).

## Conclusion and Future Prospects

This review of genetic mapping experiments of bolting and flowering time in lettuce has documented the diversity of genetic scenarios that underlie the variation in timing of phase transitions in response to environmental and developmental cues. There are several underexplored areas that warrant future research.

### Major Findings

**FIGURE 2:**
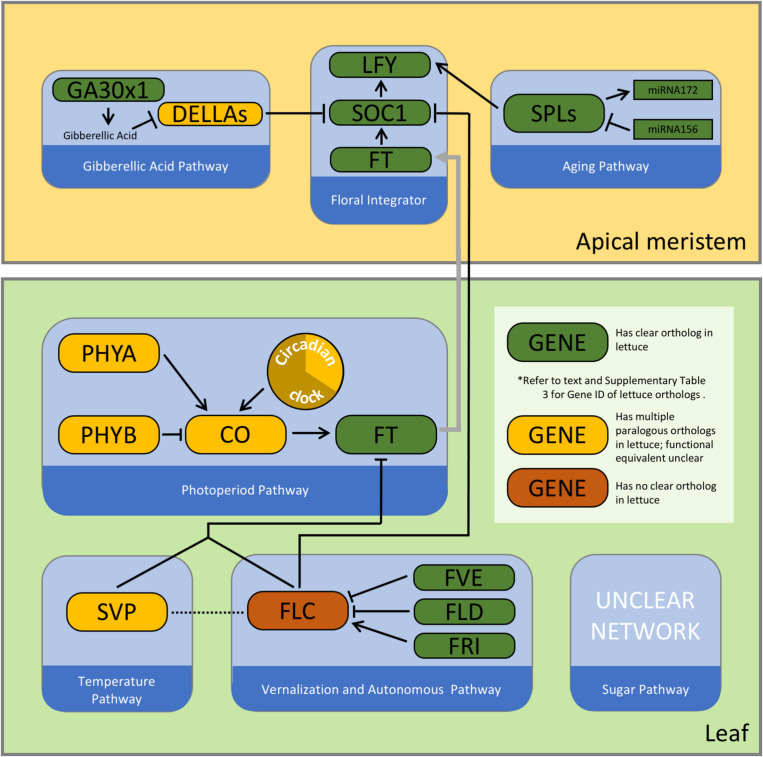
A simplified schematic of the core flowering time regulatory network in Arabidopsis. Colors of the nodes represent current information on orthology of these genes in the lettuce genome. Please refer to Srikanth and Schmid (2011); Bouché et al. (2015), and Wils and Kaufmann (2017) for examples of detailed depictions of these complex pathways. (CO: Constans; FLD: Flowering locus D; FRI: Frigida; FT: Flowering locus T; GA3OX1: Gibberellin 3-beta-dioxygenase 1; LFY: Leafy; PHYA: Phytochrome A; PHYB: Phytochrome B; SOC1: Suppressor of CONSTANS overexpression 1; SPL: squamosa promoter binding protein like; SVP: Short vegetative phase.)

1. There are many QTLs that affect bolting and flowering time in lettuce (Figure 1 and Supplementary Table 2). The phenotypic diversity in flowering time in lettuce results from a variety of genetic differences, which collocate with genes encoding different flowering time pathways. Numerous genetic mapping experiments have provided a sample of the potential genetic diversity (Supplementary Table 1). There is likely additional genetic variation affecting lettuce flowering time through other loci.
2. Despite the inevitable association of bolting and flowering time due to the developmental sequence of the two phenotypes, the genetics of these two traits was more distinct from each other than expected. Future studies should record both phenotypes to provide additional data on the genetic and molecular determinants of each.
3. The lettuce genome contains orthologs for many but not all flowering time genes identified in Arabidopsis (Figure 2 and Supplementary Table 3). Results of forward genetic, reverse genetic, and genomic analyses revealed general similarity as well as some differences in the regulation of the flowering time in lettuce compared to Arabidopsis.

4. No clear ortholog of *FLC* has been found in the lettuce genome. In contrast, there are multiple paralogs with sequence similarity to *SVP* and *CO.* Uncovering the identities of the functional equivalents of *FLC*, *SVP*, and *CO* would significantly advance our understanding of the network architecture of flowering time regulation in lettuce. Lsat_1_v5_gn_6_105061 is a potential ortholog of *SVP* due to its sequence similarity with *AtSVP* and its collocation with an intermediate-effect QTL, *qFTL6.2*. Similarly, Lsat_1_v5_gn_5_122401 could be a subject of molecular studies because of its sequence similarity to *CO*, its oscillating expression pattern that follows a 24-h period and peaks after dusk (Supplementary Table 1), and its collocation with *qBLT5.4*.
5. Ambient temperature has a major effect of both bolting and flowering. However, additional studies are needed to dissect the molecular link between heat detection and the acceleration in flowering time.
6. Less is known about the molecular basis of day-length sensitivity in lettuce because most experiments have been conducted under LD conditions. More experiments under SD conditions are needed to study the photoperiodic response of flowering time in lettuce. Such studies will facilitate the discovery of functional equivalents of *CO* in lettuce.
7. Little is currently known regarding the regulation of flowering time by sugar and starch homeostasis in lettuce. This area holds great potential because sugar and starch content could also affect the market quality of lettuce. Genetic variation in the sugar pathway could have positive or negative pleiotropic effects on quality and flowering that are important to understand.

Systems biology approaches are needed to investigate the integration of signaling pathways on genetic, transcriptional, translational, and physiological levels that result in the flowering phenotype. Previous studies have not investigated the crosstalk between pathways controlling flowering time in lettuce. Understanding the interactions between multiple environmental factors in the context of developmental regulation will be informative for guiding agricultural practices.

High throughout phenotyping and genotyping of more mapping populations and diversity panels will allow more extensive analyses on the phenotypic diversity of flowering time in lettuce. Resequencing of diverse cultivars and wild accessions combined with functional studies including genome editing will deliver a deeper understanding of flowering time regulation that can be used to address flowering-time-related breeding goals in this important vegetable crop.

## Data Availability Statement

The original contributions presented in the study are publicly available. This data can be found here: accession number DRA004561 on DNA Data Bank of Japan (DDBJ, trace.ddbj.nig.ac.jp/DRASearch/).

## Author Contributions

RM and RH conceived the study. RH conducted the literature review, performed the comparative analysis, and drafted the manuscript. MT and DL developed the mapping populations, conducted genotyping by sequencing, and performed the QTL mapping in a previously unpublished study. All authors contributed to writing the manuscript.

## Conflict of Interest

The authors declare that the research was conducted in the absence of any commercial or financial relationships that could be construed as a potential conflict of interest.

## Footnotes

1 www.flor-id.org

2 http://jgi.doe.gov/data-and-tools/bbtools/

## Supplementary Material

Supplementary Table 1 Parental lines and generation of 11 mapping populations, the number of lines, the type and number of markers, mapping software, and parameters used in the analysis, and time and location of 52 experiments reported in the review.

Supplementary Table 2 All bolting and flowering time QTLs, their physical and genetic locations, effect sizes in each experiment, flowering time orthologs within them, as well as the populations and experiments they were identified in.

Supplementary Table 3 Lettuce orthologs of genes with flowering time function in Arabidopsis, their physical locations, presence within known QTLs, the pathways they belong to, whether they are circadianly expressed in vegetative lettuce, and molecular studies that investigated their functions.
